# Chemical, Mechanical and Tribological Effects of Artificially Aging up to 6 Weeks on Virgin and Crosslinked UHMWPE Evaluated for a TKR Design

**DOI:** 10.3390/bioengineering12080793

**Published:** 2025-07-24

**Authors:** Jens Schwiesau, Bernhard Fritz, Pierangiola Bracco, Georg Bergmann, Ana Laura Puente Reyna, Christoph Schilling, Thomas M. Grupp

**Affiliations:** 1 Research & Development, Aesculap AG, Am Aesculap Platz, 78532 Tuttlingen, Germanyana_laura.puente_reyna@aesculap.de (A.L.P.R.); christoph.schilling@aesculap.de (C.S.); thomas.grupp@aesculap.de (T.M.G.); 2 Department of Orthopaedic and Trauma Surgery, Musculoskeletal University Center Munich (MUM), Campus Grosshadern, LMU Munich, 81377 Munich, Germany; 3 Department of Chemistry, University of Turin, 10125 Turin, Italy; pierangiola.bracco@unito.it; 4 Julius Wolff Institute, Charité–Universitätsmedizin Berlin, 13353 Berlin, Germany

**Keywords:** biotribology, total knee replacement, wear testing, severe conditions, aging resistance, Vitamin E stabilization

## Abstract

Patients undergo total knee arthroplasty (TKA) at younger ages with the expectation that the devices will perform well over two to three decades. During this time, the ultra-high molecular weight polyethylene (UHMWPE) bearing material properties of the implant may change due to aging induced by radiation and oxygen diffusion or other effects. Vitamin E or other antioxidants are promoted since several years to improve the oxidation resistance of UHMWPE. To compare the effectivity of these substances against established materials, a six weeks aging process was used and the chemical, mechanical and bio-tribological properties were analysed. Highly crosslinked and two weeks aged UHMWPE served as a reference for the currently established aging standards and virgin UHMWPE was aged for six weeks to separate the effects of crosslinking and vitamin E blending. Six weeks artificially aging changed the chemical, mechanical and bio-tribological properties of cross-linked UHMWPE significantly compared to only two weeks artificially aging, leading to cracks and delamination during the highly demanding activities wear test. The degradative effect of extended aging was also observed for virgin UHMWPE. These observations are in good accordance to retrieval findings. Minor changes on the chemical properties were observed for the cross-linked UHWMPE blended with vitamin E without impact on the mechanical and bio-tribological properties.

## 1. Introduction

An estimated 10% to 15% of all adults aged over 60 have some degree of osteoarthritis, with higher prevalence among women than men [1]. A common treatment for gonarthrosis is a knee arthroplasty. The knee replacement rate per 100,000 population was 137 in the OECD countries in 2019 and the number of knee replacement increased between 2009 and 2019 by 35% [2]. Average patient age for knee arthroplasty is reported for several countries between 67 and 71 years [1]. Nevertheless, patients undergo total knee arthroplasty (TKA) today at younger ages with the expectation that the devices will perform well over two to three decades of use [3,4,5,6,7,8]. The wear of the bearing components is one of the main factors influencing the long term performance of the devices [9,10,11]. Ultra-high molecular weight polyethylene (UHMWPE) was introduced in artificial orthopaedic joints as a bearing material 6 decades ago [12]. Wear resistance, oxidation resistance, and mechanical properties are the three most important material properties of UHMWPE [13]. Its chemical and microstructural properties determine the mechanical and bio-tribological behaviour. However, these properties may change during *in vivo* service due to material aging induced by radiation and oxygen diffusion or other effects [14,15,16]. Several attempts have been undertaken since the introduction of UHMWPE into the orthopaedic joint replacement to stabilize the chemical, mechanical and bio-tribological properties, e.g., packaging in inert atmosphere, crosslinking with remelting or (sequential) annealing and oxidation suppressing supplements. Furthermore, a conflict of objectives between the mechanical and bio-tribological properties exists. To maximize the implant survivorship and to minimize the need for revision, both objectives have to be balanced and the chemical properties and microstructure have to be optimized [17,18,19,20,21,22,23,24]. For hip arthroplasty, the crosslinking of UHMWPE was the major step to reduce wear and wear related osteolysis [25,26]. Nevertheless, oxidative *in vivo* material degradation of highly crosslinked UHMWPE (XPE) induced by irradiation or otherwise free radical formation is still an open topic with currently unknown clinical relevance [14,15,27,28,29,30,31,32]. Besides, the significant improvement of the clinical outcome for XPE in hip arthroplasty could not be observed in TKA up to now [33,34,35,36]. Wear and wear related aseptic loosening, which can be a result of the wear particles released by the implant bearing materials, are two complications with high impact on the long term outcome of TKA [4,10,11,37,38,39,40,41]. Reduced wear resistance of the material correlates with increased release of wear particles in the surrounding tissue of the implant and beyond. Locally, these wear particles trigger a biochemical cascade reaction involving the cytokine expression of macrophages leading to periprosthetic osteolysis. Finally, revision surgery is indicated in case of implant fixation loss. Reduced strength due to material degradation in conjunction with high loads can further lead to mechanical failure initiated by delamination, resulting in component fracture.

Vitamin E or other antioxidants are promoted since several years to improve the oxidation resistance of UHMWPE [3,42,43,44]. With these additives, the chemical reaction cascade initiated by the generation of free radicals and leading to chain scissoring, reduced crosslink density and increased crystallinity can be interrupted and the material properties are stabilized [3,18,45,46].

The aim of this study is the evaluation of the influence of cross-linking and vitamin E blending on the chemical, mechanical and tribological properties of a total knee replacement (TKR) gliding components after an extended accelerated aging duration. To simulate the long-term behaviour with regards to material degradation, the specimens were artificially aged for 6 weeks. This aging duration was applied due to previous findings, where a six weeks aging period was necessary to induce a relevant level of oxidation on highly cross-linked polyethylene [47,48]. The chemical, mechanical and tribological properties were analysed for virgin UHMWPE (GUR1020), highly crosslinked UHMWPE (GUR1020X) and highly crosslinked UHMWPE blended with 0.1% vitamin E (GUR1020XE). Highly crosslinked and two weeks aged UHMWPE (GUR1020X2w) served as a reference for the currently established aging standards (ASTM F2003 [49]). Virgin material was aged for six weeks to compare the effects of initially radical free material to material were radicals are remove after crosslinking by thermal treatment and vitamin E blending. To analyse the influence of the extended aging duration on the chemical and mechanical properties, already established methods are applied. The tribological evaluation was based on a wear simulation of daily patient activities. The ability of time-lapse simulation of highly demanding activities to generate clinically relevant wear behaviour of UHMWPE gliding components was previously reported several times [50,51,52,53,54,55,56,57,58]. In a former study [59], delamination was generated on conventional and two weeks aged UHMWPE using this method.

## 2. Materials and Methods

The tests were performed using medium sized (T3) UHMWPE gliding surfaces with a nominal thickness of 10 mm. A cruciate ligament retaining design (Columbus^®^ CR TKR, Aesculap AG, Tuttlingen, Germany) was used. The femoral component of this design has a width of 66.5 mm in the frontal plane and a length of 60.5 mm in the sagittal plane (size F4 Left). Tibial component of the same design was used (Columbus^®^ size T3) for gliding surface fixation. All metal components were of cast CoCr29Mo6 alloy [60]. GUR 1020 was blended with 0.1% vitamin E for the specimen group GUR1020XE. The effectiveness of this vitamin E concentration to supress oxidation related effects regarding the degradation of mechanical and wear properties was shown in the context of previous studies [48,51,59,61,62]. For the remaining test groups, GUR 1020 resin without additives was used. Bulk material was prepared by compression moulding.

Crosslinking was performed at 100 °C for the specimen group GUR1020X2w and GUR1020X by Gamma irradiation and for specimen group GUR1020XE by E-beam irradiation. The radiation dose was slightly increased for the vitamin E blended specimen group to compensate vitamin E related effects [63]. Specimen group GUR1020 was not irradiated. Aging of the specimens was performed according to ASTM F2003 [49] with 5 bar oxygen pressure at 70 °C for two or six weeks. After machining, artificially aging and all subsequent tests the specimens were sealed under vacuum in oxygen impermeable bags and stored at room temperature. The storage time between production and artificially aging was at least 2 month [64].

Details of each test group are listed in Table 1. Five specimens for each specimen group were aged in parallel. Four specimens were subsequently used for the wear test. To separate the influence of wear testing from mechanical and chemical analysis sections, the fifth specimen from each group was used to determine the oxidation, cross - link density and to prepare the small punch test specimens [65].

To evaluate the level of oxidation after the artificial aging process, the oxidation index (OI) was measured across the samples from the surface at 5 cutting depth up to 0.5 mm depth in 0.1 mm increments and at a cutting depth of 1.0 mm according to ISO 5834-4:2005 [59,67] by Fourier transform infrared spectroscopy (FTIR; Perkin Elmer Spectrum Image-Spotlight 200, Rodgau, Germany).

Cross-linking density and molecular mass between crosslinking were calculated based on swell ratio determination with a method derived from ASTM F2214 [68] and published by different research groups [17,27,31,32,69,70,71,72]. A minimum of 3 cylindrical pins with a diameter of 3 mm where prepared from bearing surfaces and immersed in xylene at 130 °C for 2 h. The swell ratio was calculated after conversion of the weight to a volume from weight of the specimens before and after immersion assuming a UHMWPE density of 0.935 g/cm.

Mechanical properties of the materials after artificial aging were determined by small punch testing according to ASTM F2977 [65]. Toughness and ductility are determined by this method under multiaxial loading conditions. The load–displacement–curve was evaluated regarding work to failure, displacement at ultimate load, ultimate load (load at failure) and peak load (load at the first maximum in the load–displacement–curve). The method is intended to examine relationships between wear performance and mechanical behaviour. The data were acquired with a testing speed of 0.5 mm/min on a universal testing machine (Z010, ZwickRoell, Ulm, Germany).

To avoid superimposition of wear by hydration [53] the UHMWPE gliding components for the wear simulation were soaked in test lubricant at 37 °C for a minimum of 30 days. The wear simulation was performed for a left side knee implant on a load controlled 4 station knee wear simulator (EndoLab GmbH, Riedering, Germany) capable of reproducing loads and movement of highly demanding daily activities. The performance characteristics of the set up were described previously [51,54,55]. Briefly, the load distribution and the simulation of the surrounding structures in an anterior cruciate ligament sacrificed knee is adapted from ISO 14243-1:2009 [66]. The load profiles were applied in a loop consisting of 5 frames with 4000 cycles of stair descent, 4000 cycles of stair ascent, 200 cycles of deep squatting, 1000 cycles of level walking and finally 800 cycles of sitting and rising from a chair. The loop was repeated 500 times during the test. The influence of inter–station kinematic variability was minimized by swapping the specimens during the test between the test stations [73]. The enhanced application of high flexion activities in relation to the outcome of studies of patient activities [74,75] enabled a simulation of approximately 30 years in an average knee arthroplasty patient [51]. Environmental conditions were adapted from the actual wear testing standard for TKR [66]. Newborn calf serum was diluted with deionized water to a protein content of 20 g/L. Ethylene diamine tetra acetic acid (EDTA) and Amphotericin B were added to stabilize the fluid against precipitation of calcium phosphate and microbiological contamination, respectively. The fluid temperature was 37 °C during the test. The fluid was changed every 500,000 cycles. To evaluate the wear behaviour, the weight of the UHMWPE gliding surfaces was measured after 0.5, 1.0, 2.0, 3.0, 4.0 and 5.0 million cycles. The tests were stopped after five million cycles or the destruction of one or more specimens from a test group.

An analytical balance with an accuracy of 0.01 mg was used (CPA225D, Sartorius, Göttingen, Germany). Weight drop, soak behaviour and air buoyancy were included to calculate the wear rate based on ISO 14243-2:2009 [76].

The gliding surfaces were optically evaluated with a digital microscope (Keyence VHX 5000, Neu-Isenburg, Germany). The optical evaluation was focused on the femoral articulation side of the UHMWPE gliding surfaces. Worn areas on the proximal bearing of the gliding components were indicated prior photo documentation. Description of the wear pattern were adapted from Harman et al. [77].

To evaluate the geometrical changes during the test, the specimens were scanned before the test and after 5.0 million test cycles with a 3D measuring machine with a resolution of less than 3.5 μm (UMM850, Zeiss Oberkochen, Germany). At each scan, a minimum of 7500 points on an equidistant grid covering the bearing areas of the gliding components were recorded. The scans were superimposed and the geometrical changes were calculated (Holos NT 2.4.12, Zeiss Oberkochen, Germany). The results are displayed in pseudo-colours in a plane transversal view. The geometrical changes are cumulative deformations resulting from wear and creep of the bearing materials.

Particle analysis was performed after the acid digestion of the test medium according to the method described by Niedzwiecki et al. [78]. Filter with a pore size of 20 nm were used to separate the particles. After drying and sputter coating the filter, 10 randomly selected images were taken by SEM (EVO 50, Zeiss, Germany) at 1000- and 10,000 times magnification. Equivalent circle diameter (ECD) was calculated according to ASTM F1877 [79]. Serum of the three tested samples from each specimen group was analysed after 0.5, 1.0, 2.0 and 5.0 wear cycles were applicable by image analysis (Leica QWin, Wetzlar, Germany). Image analysis results for each specimen group were averaged at each test interval.

For statistical analysis of the different test groups for all reported parameters an ANOVA was carried out. Prior to the analysis the normal distribution (p-p plots) and the homogeneity of variance (Levene test) was verified, followed by a post-hoc test (Scheffe Test) for the pairwise comparison of the parameters for the different groups. For all parameters except OI between the groups normal distribution and homogeneity of variance was given. Therefore, a non-parametric ANOVA (Kruskal-Wallis ANOVA) was performed only for OI between the groups. The level of significance was set to *p* < 0.05 for all evaluations.

For the statistical analysis, a commercial software package Statistica R13 (TIBCO Software Inc., Palo Alto, CA, USA) was used.

## 3. Results

**Figure 1:**
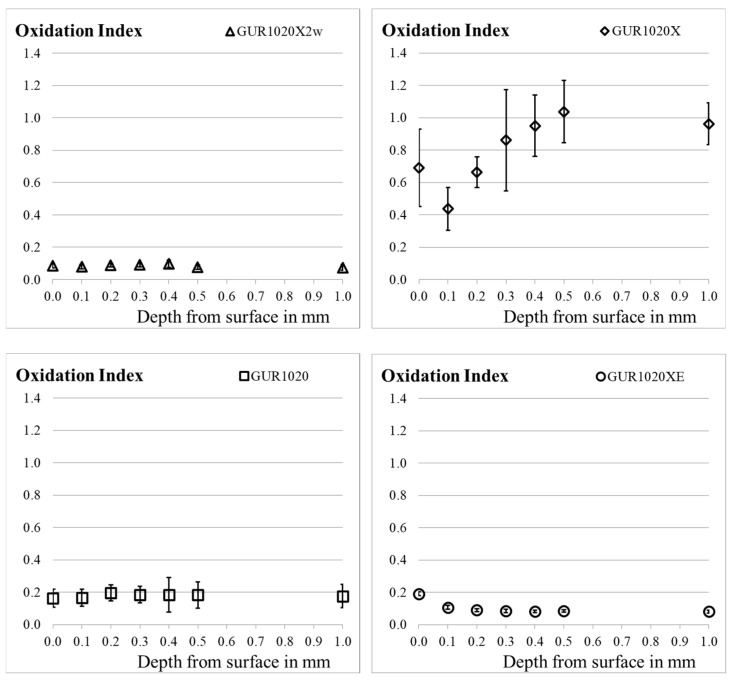
OI after two (GUR1020X2w) respectively six weeks (GUR1020X, GUR1020, GUR1020XE) of artificial aging of the gliding surfaces. Data points represent mean (±standard deviation) of 10 individual measurements.

The depth profile of the oxidation level down to 1 mm is shown in Figure 1. For specimen group GUR1020X2w, GUR1020, GUR1020XE the OI was close to or below 0.2. The OI values for specimen group GUR1020X are between 0.44 and 1.04 with an initial drop and a stable slope afterwards during the first 0.5 mm depth and no further change at 1.0 mm depth. After artificial aging the OI for the bulk for GUR1020X is higher compared to GUR1020 and both have a higher OI of the bulk than GUR1020X2w and GUR120XE. The significant differences between the specimen groups are summarized in Table 2.

Within the specimen groups no significant differences (*p* < 0.05, ANOVA, see Table 3) between OI of the different depth was observed for specimen group GUR1020 and GUR1020X2w. For specimen group GUR1020XE, the OI on the surface 0.19 (0.01) was significant above the OI measured deeper in the sample. After an initial drop of the OI from 0.69 (0.24) to 0.44° (0.13), specimen group GUR1020X revealed a significant higher OI in in the bulk.

**Figure 2:**
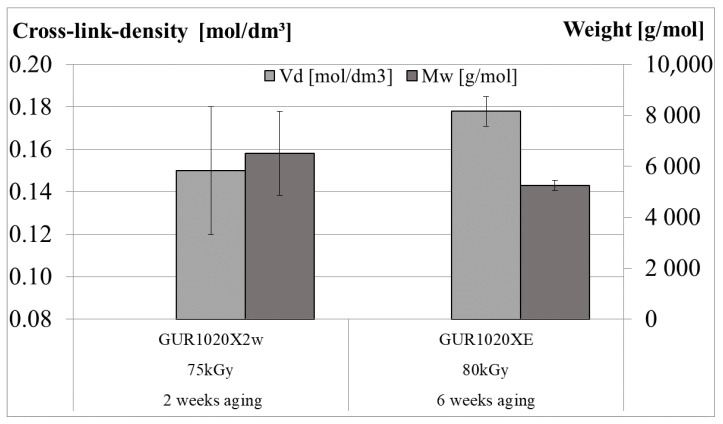
Cross-link-density (Vd) and molecular weight between cross-links (Mw), after two (GUR1020X2w) respectively six weeks (GUR1020XE) of artificial aging of the gliding surfaces. The results for specimen groups GUR1020X and GUR1020 are below the detection limit (0.08 mol/dm^3^). Data points represent mean (±standard deviation) of 3 individual measurements.

Molecular weight between cross-links and cross-linking density (a molecular volume) are visualized in Figure 2. Only for the specimen groups GUR 1020X2w and GUR1020XE values for the cross-link density could be detected. For the two remaining groups the values were below the detection limit. No significant (*p* < 0.05, ANOVA) difference was observed between the two results.

**Figure 3:**
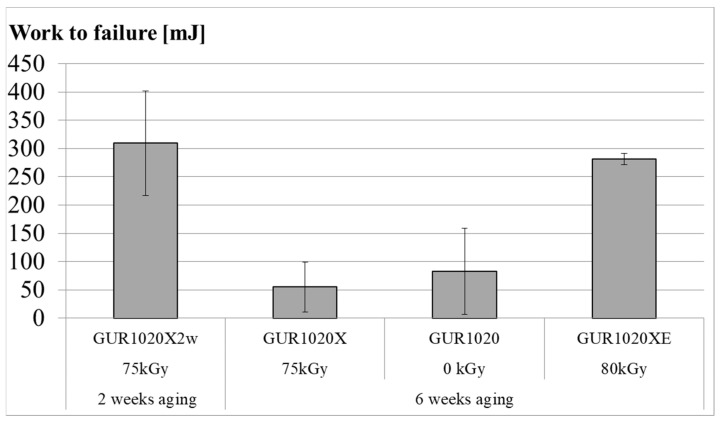
Work to failure after two (GUR1020X2w) respectively six weeks (GUR1020X, GUR1020, GUR1020XE) of artificial aging of the gliding surfaces. Data points represent mean (±standard deviation) of 6 individual measurements.

**Figure 4:**
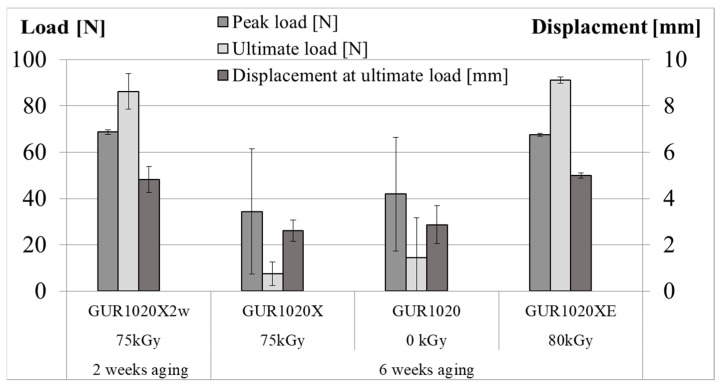
Peak load, ultimate load and displacement at ultimate load after two (GUR1020X2w) respectively six weeks (GUR1020X, GUR1020, GUR1020XE) of artificial aging of the gliding surfaces. Data points represent mean (±standard deviation) of 6 individual measurements.

Figure 3 shows results for work to failure for the individual specimen groups. Average work to failure (±standard deviation) was 309 (92) mJ for specimen group GUR1020X2w, 55 (44) mJ for specimen group GUR1020X, 83 (76) mJ for specimen group GUR1020 and 281 (10) mJ for specimen group GUR1020XE. The trend is similar for peak load, ultimate load and displacement at ultimate load (Figure 4). Average values for the peak load (±standard deviation) were 69 (1) N for specimen group GUR1020X2w, 34 (27) N for specimen group GUR1020X, 42 (25) N for specimen group GUR1020 and 68 (1) N for specimen group GUR1020XE. The average ultimate load (±standard deviation) values are 86 (8) N for specimen group GUR1020X2w, 8 (5) N for specimen group GUR1020X, 14 (17) N for specimen group GUR1020 and 91 (1) N for specimen group GUR1020XE. Displacement at ultimate load is a value for ductility. Average values (±standard deviation) are 4.8 (0.6) mm for specimen group GUR1020X2w, 2.6 (0.5) mm for specimen group GUR1020X, 2.9 (0.8) mm for specimen group GUR1020 and 5.0 (0.1) mm for specimen group GUR1020XE. Higher ultimate load compared to peak load indicates a strain hardening effect [80]. This effect was observed for specimen groups GUR1020X2w and GUR1020XE (Figure 4).

Several significant differences were observed for the mechanical properties between the individual specimen groups, these are summarised in Table 4.

**Figure 5:**
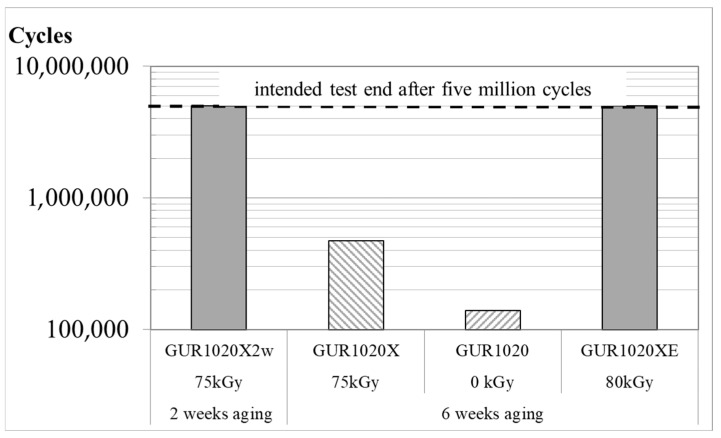
Numbers of wear cycles reached during the individual test. Only the unshaded bars represent tests reaching the intended five million cycles. The logarithmic scaling should be noted.

**Figure 6:**
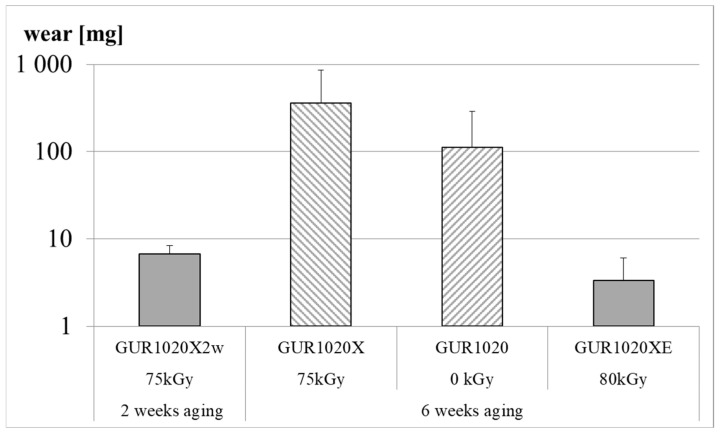
Cumulative wear of the gliding surfaces by the end of each test. Bars indicate mean (±standard deviation). Only the unshaded bars represent tests reaching the intended five million cycles. The logarithmic scaling should be noted.

**Figure 7:**
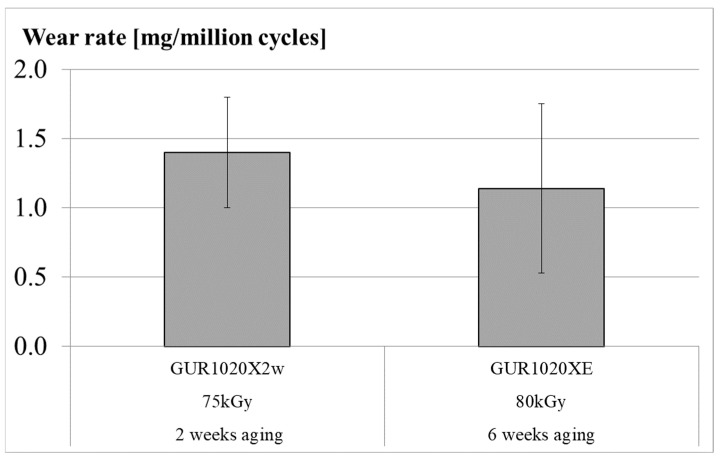
Gravimetric wear rate (mean ± standard deviation) for the specimens of the two tests reaching the intended five million cycles. According to ISO 14243-2 [76], the calculation of the wear rate starts at 0.5 million cycles to avoid the effect from the running in wear.

Wear was gravimetrically detected up to five million cycles or until failure of one or more specimens from a specimen group. The intended five million wear cycles were reached by specimen group GUR1020X2w and GUR1020XE (Figure 5). Cracks and subsurface cracks were noticed after 471,800 wear cycles for specimen group GUR1020X on two specimens (Figure 5 and Figure 10). Fracture of one specimen and subsurface cracks on three specimens was detected after 140,000 wear cycles for specimen group GUR1020 (Figure 5 and Figure 11). At the end of the test, the cumulative gravimetrical wear (±standard deviation) was 6.7 (1.6) mg for specimen group GUR1020X2w, 358.2 (503.4) mg for specimen group GUR1020X, 111.3 (177.9) mg for specimen group GUR1020 and 3.3 (2.7) mg for specimen group GUR1020XE (Figure 6). No significant difference was detected between the individual specimen groups for the cumulative wear. The wear rate (±standard deviation) for the two specimen groups reaching five million wear cycles were 1.3 (0.4) mg/million cycles for GUR1020X2w and 1.1 (0.6) mg/million cycles for specimen group GUR1020XE (Figure 7). The difference of the wear rate between specimen group GUR1020X2w and specimen group GUR1020XE was not significant.

**Figure 8:**
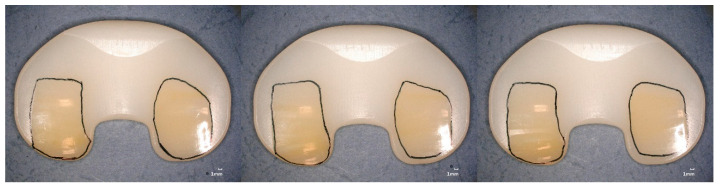
Proximal surface of the tested specimen GUR1020X2w after five million cycles of highly demanding activities. Worn areas indicated by scratching, polishing and striated pattern are framed, scaling is 1 mm.

**Figure 9:**
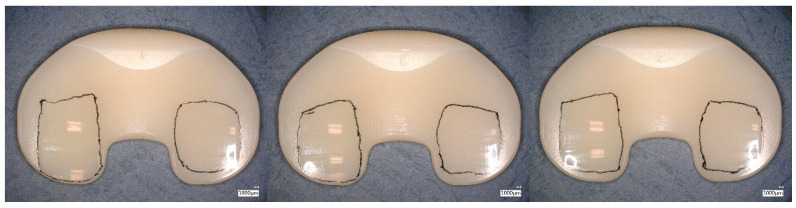
Proximal surface of the tested specimen GUR1020XE after five million cycles of highly demanding activities. Worn areas indicated by scratching, polishing and striated pattern are framed, scaling is 1 mm.

**Figure 10:**
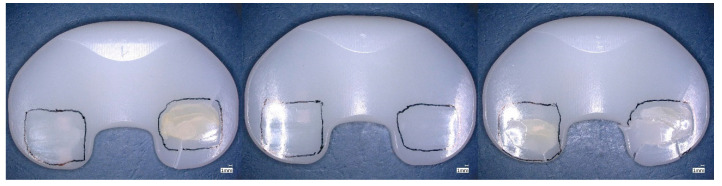
Proximal surface of the tested specimen GUR1020X after 471,800 cycles of highly demanding activities. Worn areas indicated by cracks (left and right specimens), subsurface cracks (left and right specimens), scratching, polishing and striated pattern are framed, scaling is 1 mm.

**Figure 11:**
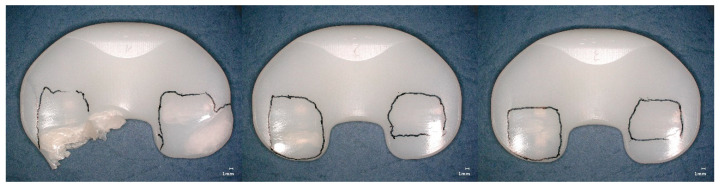
Proximal surface of the tested specimen GUR1020 after 140,000 cycles of highly demanding activities. Worn areas indicated by fracture and delamination (left specimen), subsurface cracks, scratching, polishing and striated pattern are framed, scaling is 1 mm.

Burnishing was dominant in the worn regions for the tested specimens reaching five million wear cycles (specimen group GUR1020X2w, Figure 8 and GUR1020XE, Figure 9). Cracks and subsurface cracks were observed on two specimens of specimen group GUR1020X, all specimens of this specimen group show scratching, polishing and striated pattern (Figure 10). Specimen group GUR1020 shows fracture and delamination on one specimen, the remaining specimens indicate signs of subsurface cracks and abrasive wear (scratching and burnishing) (Figure 11). The worn areas of the two specimen groups reaching five million cycles (GUR1020X2w and GUR1020XE) are larger than the worn areas of the specimen groups with a premature test end.

**Figure 12:**
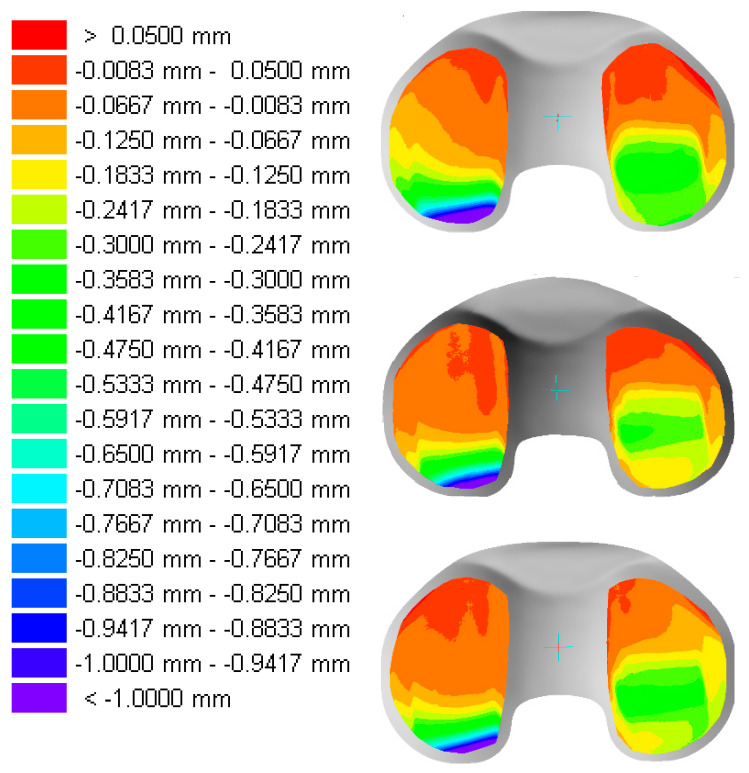
Geometrical changes of the proximal surface of the tested specimen GUR1020X2w after five million cycles of highly demanding activities. left = lateral, right = medial, Scale: red > 0.05 mm and purple < 1.00 mm.

**Figure 13:**
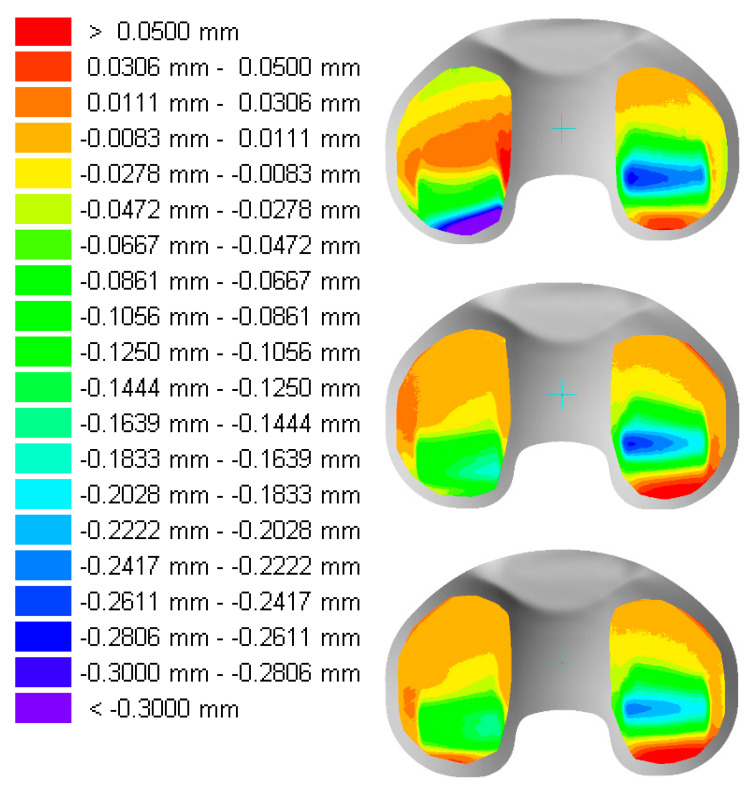
Geometrical changes of the proximal surface of the tested specimen GUR1020XE after five million cycles of highly demanding activities. left = lateral, right = medial, Scale: red > 0.05 mm and purple < 0.30 mm.

The deformation of the samples from specimen group GUR1020X2w and GUR1020XE are presented in Figure 12 and Figure 13. It should be noted that a different colour spread is used for the two different specimen groups. The position of maximal deformation is on the lateral side in the posterior region of the bearing and on the medial side close to the dwell point (deepest position of the concave bearing in the sagittal cross section) on the specimens from specimen group GUR1020X2w. The deformation was more pronounced on the lateral side with maximum lateral deformation of 1.0 mm and more and medial maximum deformation of 0.475 mm. For specimen group GUR1020XE the deformation was more equally distributed with maximum lateral deformation between 0.20 mm and 0.30 mm and a maximum medial deformation between 0.22 mm and 0.28 mm. A posterior shift of the maximum deformation on the lateral side compared to the medial side can be noticed for all specimens.

**Figure 14:**
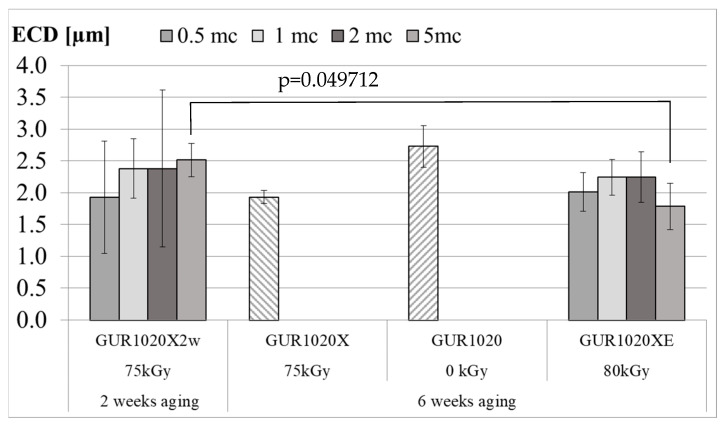
Average ECD (±standard deviation) for the selected test intervals mc (million cycles). Only the unshaded bars reached the intended number of cycles for the test interval. Indicated is the only significant difference.

The particle size (Figure 14) was calculated based on the size of an equivalent circle particle diameter (ECD). For the cross-linked and two weeks aged material (GUR1020X2w) the average particle size (±standard deviation) changed from 1.93 (0.88) µm at 0.5 million wear cycles to 2.38 (0.47) µm, 2.38 (1.24) µm and 2.51 (0.27) µm at 1.0, 2.0 and 5.0 million wear cycles, respectively. The same cross-linked material aged for six weeks (GUR1020X) had a particle size of 1.93 (0.10) µm after 471800 wear cycles. Six weeks aged material without crosslinking (GUR1020) had an average particle size (±standard deviation) of 2.73 (0.33) µm after 140,000 wear cycles. For specimen group GUR1020XE the average particle size (±standard deviation) alternates around 2 µm with values of 2.01 (0.31) µm at 0.5 million cycles, 2.24 (0.28) µm at 1.0 million cycles, 2.24 (0.40) µm at 2.0 million cycles and 1.79 (0.37) µm at 5.0 million cycles.

## 4. Discussion

The aim of this study was the evaluation of the influence of cross-linking and vitamin E blending on the chemical, mechanical and tribological properties of TKR gliding components after an extended accelerated aging duration. A reference group (GUR1020X2w) with cross-linked and two weeks accelerated aged gliding component material was used. In this discussion the results are compared to retrievals as far as possible to assess the applied method to predict clinical findings.

The oxidation index (OI) is a predictor regarding aging and subsequent degradation of mechanical properties in UHMWPE [22,23,42,70,81,82]. Oxidation is a chemical driven process initiated by free radicals in the UHMWPE leading to the scission of the molecular chains [3,32,83]. The threshold value of OI for UHMWPE is reported in abroad range between 0.25 and 3 [19,20,23,81,84,85,86,87,88]. This is related to different analytical methods used to identify the oxidation (evaluation of the ketone or carbonyl peak), different mechanical property used as target value (e.g., ultimate tensile strength, work to failure or delamination) and different correlation between mechanical properties and oxidation for different modifications of UHMWPE [86].

The virgin UHMWPE (GUR1020) is expected to be radical free before accelerated aging, similar to not cross-linked and ethylene oxide (EtO) or gas plasma (GP) sterilized UHMWPE as these two sterilisation methods do not change the physical, chemical and mechanical properties of UHMWPE substantially [89]. The influence of artificial aging on the mechanical degradation behaviour of non irradiated UHMWPE with negligible OI was previously reported by Edidin et al. [64] and was proposed as a treatment in the context of wear simulation [90]. For not cross-linked and ethylene oxide (EtO) or gas plasma (GP) sterilized material, low surface oxidation was reported for the new material and any oxidation was related to mechanical degradation [91]. In a subsequent study on retrievals, oxidation was reported and related to poorly consolidated material weakened by stresses initiated during the production process [92]. Unfortunately, due to the applied analytical method, these results can be compared only in a qualitative way and they are incomparable to the OI value detected according to ISO 5834-4 [67] and ASTM F2102 [93]. Regis et al. [16] reported OI values (ketone peak evaluation with reference band at 2020 cm^−1^ and recalculated to OI) of 0.4–0.8 in the high loaded region for EtO sterilized THA acetabular insert retrievals after up to sixteen years *in vivo*. This is two to four times higher compared to the OI values observed in this study for virgin UHMWPE accelerated aged for 6 weeks (GUR1020) and may be attributed to the use of calcium stearate as processing aid in the retrieval material. In contrast, the unirradiated and EtO or GP sterilized TKR and THR retrievals implanted for up to 10 years reported by MacDonald et al. [94,95], the EtO or GP sterilized hip liners implanted up to 25 years reported by Rowell et al. [88] and the EtO sterilized and not irradiated tibial inserts implanted up to 17 years reported by Williams et al. [96] are in a range of OI values comparable or below to the results of specimen group GUR1020. This is also confirmed by Oonishi et al. [97] for the unloaded region of an initially EtO sterilized retrieval after 23 years of implantation. Nevertheless, the retrieved inlay showed a tendency of higher OI (0.45–1.83) at heavier loaded positions. No oxidation of EtO sterilized tibial inserts after shelf storage up to 19 years is reported by Currier et al. [19]. In the same study, the oxidation of retrievals correlated with implantation time and the average oxidation was in a range comparable to specimen group GUR 1020. Nevertheless, individual OI (ketone oxidation index) up to 2.5 are reported. The influence of mechanical stresses on the initiation of oxidation in non irradiated material is highlighted in the studies reporting higher OI [16,19,92,93,94,97]. The use of an unloaded reference for the OI quantification to specify the oxidation of specimen group GUR 1020 seems to be the most reasonable explanation for the difference in the OI between some retrieval results and the results for specimen group GUR 1020. Further, the sampling position within the retrievals to evaluate the oxidation with regards to the loading conditions are a further source for inconsistencies between the experimental specimens and the retrievals.

The lowest oxidation (≤0.1) was observed for the two weeks accelerated aged highly crosslinked specimen group GUR1020X2w. This result is in agreement to previous publications regarding artificial aging of XPE [13,48,86]. A significant change in the OI was observed for this material after six weeks of accelerated aging (GUR1020X). This corresponds to observations by Fung et al. [86] and Grupp et al. [48] where no detectable OI was observed after accelerated aging of crosslinked material with subsequent remelting up to 4 weeks but at 6 weeks the oxidation was noticed. For specimen group GUR1020X, OI values up to 1.0 were observed, which is in the range reported as a threshold value corresponding to a decrease of mechanical properties below standard requirements for conventional UHMWPE [81,85]. A similar maximum OI (ketone peak evaluation) was reported by Currier et al. [14] and Reinitz et al. [32] for a 3 times Gamma irradiated tibial insert receiving a cumulative radiation dose of 90 kGy with subsequent annealing below melt temperature and more than 3 years *in vivo*. In the study by Reinitz et al., material with lower irradiation and subsequent remelting of the UHMWPE was analysed as well and revealed lower OI. For all tested materials in this study the OI correlated to the implantation time. The reduced oxidation for lower irradiation and subsequent remelting of the UHMWPE was confirmed by a study of Liu et al. [31]. Furthermore, the increase of oxidation in loaded versus unloaded regions was reported. A threshold level for the OI around 3 was established for 50 kGy e-beam cross-linked material by Medel et al. [87]. In this study, delamination was observed only for cross-linked and annealed (below the melting point) GUR1050 after consecutive aging (120 °C in air for 36 h) and mechanical loading steps. For specimen group GUR1020X, the depth profile for the OI revealed an increasing OI up to a depth of 0.5 mm. To the authors knowledge, an increasing OI with increasing depth after an artificially aging process according to the current ASTM standard [49] for six weeks is reported for the first time for cross-linked UHMWPE. This behaviour replicates OI depth profiles for retrievals [14,23,71] and was previously reported for cross-linked and remelted UHMWPE after 10 weeks artificially aging with a reduced temperature [86].

The initiation of oxidation of UHMWPE can be supressed by stabilizing substances like vitamin E [18,23,28,46,63,85,92,98,99,100,101,102]. This effect can be observed for specimen group GUR 1020XE. After six weeks of accelerated aging, the OI profile shows decreasing values down to 0.3 mm, reaching a stable OI of the bulk material below 0.1. On the surface, the OI value was increased but still in a range without clinically significance. An increase of the OI near the surface was also observed on retrievals containing antioxidants in two studies by Currier et al. [23,103]. Ponzio et al. reported similar observations for retrieved tibia inserts (gliding surfaces) out of remelted highly crosslinked UHMWPE and antioxidant-stabilized highly crosslinked UHMWPE [21]. Rowell and Muratoglu [104] reported a similar behaviour for vitamin E-stabilized, crosslinked UHMWPE implants after short-term *in vivo* service evaluating the carbonyl index, stating that unextractable carbonyl groups are believed to be predominantly associated with polymer-derived oxidation products. They conclude that the carbonyl profile surface maximum appears to be the consequence of mechanisms unrelated to *in vivo*-initiated oxidation and do not appear to increase with either *in vivo* duration or total lifetime of the implant. In two publications of Currier et al. [84,103] the surface maximum OI was related to absorbed species from the *in vivo* environment. The second explanation for the surface maximum of the OI is not applicable to our results as the specimen was nor immersed in (simulates) body fluid. Spece et al. [85] reported the largest retrieval group of highly crosslinked UHMWPE with vitamin E up to now. There the implantation time positively correlates with the OI of the articulating surface. In this study, the average OI for retrievals containing vitamin E was below 0.1 in all analysed regions. Nevertheless, individual OI values up to 0.4 are reported even for short implantation times. The same author reviewed the current literature regarding the *in vivo* performance of antioxidant stabilized UHMWPE in TKR, no critical raise of the OI was reported.

Oxidation is accompanied by a reduction of the cross-link density of initially cross-linked (annealed and remelted) UHMWPE [23,31,32,71]. Cross-link densities of 0.19 mol/dm^3^ and 0.21 mol/dm^3^ for the loaded and unloaded regions, respectively, of retrieved gliding components are reported by Liu et al. [31] for variations of cross-linked UHMWPE irradiated with 50 kGy to 75 kGy and subsequently remelting as well as sequentially irradiated and annealed with a cumulative irradiation dose of 90 kGy. Surprisingly, these values are well above the cross-linked density expected based on the studies by Muratoglu et al. [17] and Oral et al. [24] comparing the irradiation doses and the cross-link density. This observation is confirmed by Reinitz et al. [32,71], Currier et al. [23] and Ponzio et al. [21] who reported similar cross-link densities for the same materials like Liu et al. A decrease of the cross-link density with implantation time is reported by Reinitz et al. for sequentially irradiated and annealed retrievals [32] and for retrievals remelted after cross-linking [71], which may explain the undetectable crosslink density for the cross-linked material without vitamin E after 6 weeks artificially ageing (GUR 1020X). Nevertheless, the pathway for the reaction in the retrievals is explained based on diffusion of reactive molecules from the joint fluid, which can not be applied to specimens artificially aged only in oxygen. A further correlation between cross-link density and transvinyl index is reported by Currier et al. [23] for short term retrievals of cross-linked and remelted UHMWPE and for cross-linked UHMWPE treated with antioxidants (blended and diffused) which can be linked to irradiation dose sensitivity of both parameters [17,105]. The cross-link density for antioxidant containing cross-linked UHMWPE in this study has shown no correlation to the oxidation and had a range between 0.17 mol/dm^3^ and 0.25 mol/dm^3^. This range covers the results for cross-link densities of retrievals made from vitamin E diffused UHMWPE reported by Rowell et al. [104] and retrievals made from vitamin E blended and diffused UHMWPE reported by Ponzio et al. [21]. Cross-linking was not detectable for the virgin material (GUR 1020) and the crosslinked material without vitamin E after six weeks aging (GUR 1020X). For the virgin material this corresponds to the intended material properties. In the cross-linked material, cross-linking is no longer detectable only after extended aging. The same material after two weeks aging (GUR1020X2w) had a crosslink density of 0.15 mol/dm^3^ ± 0.03 mol/dm^3^, whereas the crosslinked material with 0.1% initial vitamin E had a crosslink density of 0.18 mol/dm^3^ ± 0.01 mol/dm^3^ after six weeks aging. These values are comparable to crosslink densities reported by Muratoglu et al. [17], where a crosslink density of 0.15 mol/dm^3^ corresponds to an absorbed radiation dose between 40 and 50 kGy and a crosslinked density of 0.18 mol/dm^3^ to an absorbed radiation dose around 80 kGy. These values indicate a change in the crosslink density for specimen group GUR 1020X2w compared to the initial irradiation dose related cross-link density, but not for specimen group GUR 1020XE. Nevertheless, these conclusions should be interpreted with caution as there are some differences in the materials used by Muratoglu et al. and a later study by Fung et al. [86] revealed higher cross-link densities at radiation doses used in both studies.

Muratoglu et al. and Fung et al. also evaluated the mechanical properties for a broad range of materials [17,86]. Unfortunately, their test method differs from the method applied in our study due to the different specimen sources (bars, rods or plates vs. final products). Nevertheless, qualitative information can be transferred between the test methods due to some correlation [106,107] and will be discussed in the following section. Early results according to the method in our study are reported by Kurtz et al. [80,107,108]. Chemical crosslinked GUR 1020 is reported with a peak load around 76 N ultimate load around 82 N, displacement at ultimate load around 4.1 mm and work to failure around 229 mJ [80]. The peak load is approximately 10% above the values for specimen group GUR1020X2w and GUR1020XE, whereas the ultimate load is 5% below the values for specimen group GUR1020X2w and 11% below for specimen group GUR1020XE and the displacement at ultimate load approximately 12% below the values for specimen group GUR1020X2w and GUR1020XE. The work to failure for specimen group GUR1020X2w is 35% and for specimen group GUR1020XE 22% above the values reported by Kurtz et al. [80]. This indicates no substantial influence of the aging process on the mechanical properties of specimen group GUR1020X2w and GUR1020XE. For specimen group GUR1020XE this conclusion is confirmed by Mulliez et al. [109]. Nevertheless, the values for specimen group GUR1020X are reduced substantially compared to the report by Kurtz et al. [80], indicating the impact of the extended aging on the mechanical properties of this material. This is also observed for specimen group GUR1020. Results of a small punch test on nonirradiated GUR1020 after 2 weeks aging similar to our method reported by Chiesa et al. [110] revealed no relevant effect, but tensile testing showed reduced ultimate tensile strength after aging. Non-irradiated UHMWPE with higher molecular weight than GUR 1020 was not affected at 80 °C in air [108] and GUR 1020 was not affected at 63 °C in oxygen at 5 atm [86] after accelerated aging up to 4 weeks. The later study confirmed our observation on virgin GUR1020 that mechanical properties are reduced after 6 weeks aging whereas the OI shows only small changes. 5% lower ultimate load was observed for non-irradiated tibia bearing retrievals with an implantation time up to 10 years compared to non-implanted tibial bearings of the same design. Additionally, for the subsurface region, a negative correlation between implantation time and work to failure is reported in the study of MacDonald et al. [94]. The work to failure of the retrievals in this study is still three times above the values recorded after 6 weeks aging for specimen group GUR1020. About 20% lower work to failure (toughness) was reported by Oonishi et al. [97] for the unloaded region of a retrieval after 23 years of implantation compared to the retrievals analysed by MacDonald et al. [94]. Moreover, the trend for higher OI at heavier loaded position was also reflected in lower work to failure (toughness). Currier et al. [19] measured the mechanical properties for non-irradiated UHMWPE tibia bearings from stock and retrievals in an uniaxial tensile test, revealing for the retrievals a higher yield stress and a decline of the ultimate stress with higher oxidation similar to virgin GUR1020 after long term accelerated aging [86]. The ultimate tensile stress correlated inversely with the oxidation in an uniaxial tensile test for tibia bearings crosslinked with 65 kGy and subsequent remelting retrieved after up to 3 years of implantation, this correlation was not observed for tibial bearings containing vitamin E with a comparable implantation time [23]. This trend is similar for specimen group GUR1020X and GUR1020XE and confirmed by small punch tests on vitamin E infused GUR 1020 crosslinked with 100 kGy and subsequent gamma sterilisation (25 kGy–40 kGy) after aging up to 4 weeks [111].

For sequentially irradiated and annealed highly cross-linked UHMWPE gliding components, a work to failure below 200 mJ is reported to be indicative for oxidative degeneration [30]. The correlation between wear and mechanical behaviour of GUR 1050 in knee implants irradiated with radiation doses up to 200 kGy was investigated by Akagi and Asano [112,113] in wear tests simulating the stance phase of the gait and small punch testing. They showed that toughness (work to failure) of the tibia bearing material was reduced after simulator testing. The 75 kGy irradiated material reduced the initial toughness of 227 mJ to 205 mJ after the wear test. These values are approximately 25% to 50% below the value for specimen group GUR1020X2w and GUR1020XE, respectively. This effect is mainly related to a higher ultimate displacement (ductility) for specimen groups GUR1020X2w and GUR1020XE. Nevertheless, the remaining toughness (work to failure) after 6 weeks aging of the specimen group GUR1020X was only a quarter of the initial toughness measured in the study of Akagi et al. for 75 kGy irradiated GUR 1050.

During the last decades, several reports were published concerning delamination or fatigue failure of UHMWPE gliding components [9,30,82,99,114,115,116,117,118,119,120,121,122,123,124,125,126] and several tests have been prepared to simulate this failure mode [55,56,59,82,127,128,129,130,131,132,133,134,135]. Some of the retrieval reports described mid-term delamination within the first 5 years [114,115]. These results are related to first generation UHMWPE sterilized by gamma radiation in air. This failure mode was reduced by sterilization with Ethylene Oxide [91,96,99] due to the absence of irradiation degradation. However, after long *in vivo* duration, fatigue damage for never irradiated UHMWPE joint implants including gliding components from knee implants is observed [19,97]. Alternatively, irradiation sterilisation of UHMWPE in an inert atmosphere was introduced to reduce shelf life oxidation and degradation of mechanical material properties. After this adaptation, the material degradation was postponed but not avoided [119,136]. For highly crosslinked UHMWPE, reports related to retrievals with delamination or fatigue failure are rare. Derr et al. [137] reported about six highly crosslinked and sequentially annealed gliding components with fatigue failure after a mean implantation time of 7.6 years associated with overloading due to inadequate alignment of the implants. For the same material, Kop et al. [30] reported delamination or fatigue failure on five retrievals with an implantation time of 4 to 5 years. Recently, Asher et al. [9] published a delamination rate of 64% for highly crosslinked retrievals implanted for more than 6.5 years and an association between delamination and instability. Based on the chemical characteristics explained previously, material degradation is noticeable for the initially highly crosslinked retrievals. This effect is time dependent [30,31,71,137,138] and reflected by the absent of delamination for the highly crosslinked (75 kGy) test group after accelerated ageing for 2 weeks (GUR1020X2w) and the observation of fatigue failure for the 6 weeks accelerated aged test group (GUR 1020X).

Cracks, delamination and subsurface cracks as a precursor for delamination and fracture indicate fatigue as the primary failure mode for the two early failing specimen groups (GUR1020X and GUR1020, Figure 9 and Figure 10). The early failure of the specimen groups GUR1020 and GUR1020X reflects the failure mode observed on retrievals, but the onset during the wear test is shortened due to the time-laps effect of the extended accelerated aging. The destruction of specimen group GUR1020 appeared earlier and more severe compared to specimen group GUR 1020X, Base on the previous discussed results for the chemical and mechanical properties this could not be expected. The reason for this behaviour is unclear up to now and further investigations are needed to understand that behaviour. Furthermore, this observation indicates the complexity of wear and that mechanical and chemical parameters give hints but cannot substitute a wear test.

Results for a non-irradiated GUR1020 blended with 0.1% vitamin E accelerated aged for six weeks and tested under similar conditions show no delamination nor fatigue failure [62]. This observation indicates a protective effect of Vitamin E against free radicals induced in virgin UHMWPE and a stabilisation of the mechanical properties of non-irradiated UHMWPE. The in situ reaction of Vitamin E with in situ generated free radicals from molecular chain scissoring is the most likely explanation for this difference. However, further studies are necessary to proof this explanation, since current assumptions are related only to free radicals induced by cyclic loading and synovial liquid fluids [139]. The absence of delamination and fatigue failure for the vitamin E stabilized test group (GUR1020XE) after 6 weeks of accelerated aging indicates stable mechanical properties as already noticed by the small punch tests. The protective effect of the vitamin E blending regarding the mechanical degradation and subsequent delamination and fatigue failure of the UHMWPE gliding components is demonstrated for non-irradiated and highly crosslinked material and is similar to previous results for medium irradiated GUR1020 with 0.1% vitamin E [62].

The cumulative wear and the wear rate for both groups reaching the intended five million test cycles (GUR1020X2w and GUR 1020XE) are not significantly different and below a currently proposed threshold level of 3 mm^3^/million cycles (converted by density to mg/million cycles) for highly crosslinked UHMWPE. This threshold was developed by comparing results from wear simulator tests with implant registry data [6]. Osteolysis as a clinical finding associated with wear of joint implants was used by Bitsch et al. [140] to identify a critical wear rate of 15.4 mm^3^/y or 8.0 mm^3^/million cycles for a highly crosslinked UHMWPE in hip implants. After the evaluation of 53 wear tests with different types of crosslinked UHMWPE, an approximated reasonable threshold of 5 mg/million cycles is proposed by the first European Consensus of the European Federation of National Associations of Orthopaedics and Traumatology (EFORT) for hip implants [141]. Unfortunately, due to lack of data no threshold is proposed in this paper for the wear of TKR with crosslinked UHMWPE. It should be emphasised that these values are related to an average patient activity and can serve only as an orientation when comparing to the results of the highly demanding activities applied on the tested specimens in this study.

Higher deformation after five million highly demanding activities load cycles was recorded for the specimen group GUR1020X2w compared to specimen group GUR1020XE. This probably reflect the cumulative gravimetric results, even though no statistical significance can be provided for the differences due to the limited sample size. In both specimen groups, the position of the maximum deformation of the medial compartment was closer to the middle of the bearing and more posterior for the lateral compartment. This distribution of the geometrical changes is confirmed by previous tests with the same implant design and test conditions for different materials and reflects the predominance of internal torque on the tibia during the applied highly demanding activities [51,52,59]. An average *in vivo* duration of approximately 30 years of highly demanding activities is simulated in this study [54]. Based on this assumption, the average annual penetration rate can be estimated with 0.033 mm/year on the lateral side and 0.016 mm/year on the medial side for specimen group GUR1020X2w and 0.01 mm/year on both sides for specimen group GUR1020XE. The change in the geometry due to the articulation of the implant components is design specific and related to the applied kinematic conditions, finally leading to wear and creep of the bearing material. Nevertheless, some retrieval studies show geometrical changes comparable to the wear simulation results of this study. The wear analysis of the retrievals can not be based on the direct geometrical comparison before and after *in vivo* service, thus it is based on the initial product design or surface reconstruction techniques. These methods neglect the production tolerances. Furthermore, creep is part of the geometrical changes and has to be considered in the interpretation of wear [124,142]. Therefore, quantitative results should be assessed with caution and, if cited, be an orientation rather than a threshold. However, the qualitative evaluation seems reasonable. After 23 years in clinical use, a cruciate ligament sacrificing ceramic based implant with an EtO sterilized gliding component machined from GUR412 was retrieved and analysed by Oonishi et al. [97]. The geometrical changes of this retrieval were lager on the medial side compared to the lateral side. The position for the maximum deformation on the lateral side was similar to that of the wear simulation in this study, whereas the main deformation on the medial compartment was more posterior orientated. The maximum penetration depth for this retrieval is reported with 0.851 mm (estimated average annual penetration rate 0.037 mm/year) on the medial side and 0.718 mm (estimated average annual penetration rate 0.031 mm/year)on the lateral side. A volumetric wear rate of 18.8 mm^3^/year was calculated. Similar medial and lateral penetration rates are reported for 64 retrievals of an anterior cruciate retaining design made from GUR415 by Knowlton et al. [124]. A wear rate of 12.9 mm^3^/year was calculated with a medial penetration rate of 0.035 mm/year and a lateral penetration rate of 0.034 mm/year. In this study, a wide scatter is reported for the position of the maximum penetration depth, which rarely coincided with the centre or the location of minimum thickness on the gliding components. This observation may reflect the sensibility of the evaluated design on implant positioning and patient variabilities. Asher et al. [9] analysed the penetration for conventional and highly crosslinked UHMWPE retrievals with an implantation time of 11.7 ± 4 years. A medial penetration rate of 0.054 mm/year and a lateral penetration rate of 0.051 mm/year was found for conventional UHMWPE and a medial penetration rate of 0.014 mm/year and a lateral penetration rate of 0.011 mm/year for highly crosslinked material In a recent study by Valič et al. [122], the geometrical evaluation of 57 retrievals of one specific posterior cruciate ligament retaining design revised due to loosening or osteolysis shows an area and position of the geometrical changes on the medial compartment similar to specimen groups GUR1020X2w and GUR1020XE. The lateral compartment showed geometrical changes more centred compared to the simulation in this study. The calculated volumetric wear rate in the study of Valič et al. was 15.1 mm^3^/year. Finally, the analysis of over 1500 retrievals by Currier et al. [143] suggested that a gliding component with a wear rate below 0.03 mm/year to 0.04 mm/year would be unlikely to require revision for wear related reasons. Future studies related to ex vivo or *in vivo* evaluation of highly crosslinked gliding surfaces will verify or revise this conclusion [9]. The wear rate of 1.3 (0.4) mg/million cycles for GUR1020X2w and 1.1 (0.6) mg/million cycles for specimen group GUR1020XE and the average annual penetration rate between 0.016 mm/year to 0.033 mm/year for specimen group GUR1020X2w and for specimen group 0.01 mm/year for GUR1020XE is below all the previously reported values and reflects the improved wear resistance of the materials due to crosslinking.

Apart from destruction of the implant components, the physiological interaction of the surrounding tissue with the wear debris, leading to osteolysis or loosening [144,145] of implant components, plays a major role in implant failure and revision [40,41]. The biological reaction is related to the chemical composition [146,147,148], the size of the released wear particles [149,150] and the amount of wear [37,151]. The amount of wear is directly related to the cumulative volumetric or gravimetric wear and wear rate. The size of the debris must be analyzed separately. This was done after selected time points of the test. Within the specimen groups, no significant differences were detected for the average size at specific time points. Only at the end of the test, the particle size detected for specimen group GUR1020XE is smaller compared to the particle size of specimen group GUR1020X2w, with 1.79 (0.37) µm versus 2.51 (0.27) µm at 5.0 million cycles. The particles are in the biological active size range established after in vitro experiments of Green et al. [149], were murine peritoneal macrophages were stimulated by conventional polyethylene. The same group stimulated primary human peripheral blood mononuclear phagocytes with different sizes of UHMWPE (GUR412) and observed the highest biological response for particles in the submicrometric size range [152]. The size range of the specimens in all specimen groups are also similar to the mean size found by Shanbhag et al. [125] in periprosthetic tissue of knee implants made from conventional UHMWPE. Nevertheless, several other studies reported smaller particles in the surrounding tissue or fluid of knee implants. Benz et al. [153] found a mean particle length of 0.9 (0.8) µm in tissue retrieved during revision surgery of three knee implants. For different bearing designs (medial pivot, mobile bearing, posterior stabilized) and different bearing materials (conventional and crosslinked UHMWPE, CoCr, ceramic and oxidized zirconium counterparts), Minoda et al. [154,155,156,157,158] found one year postoperatively an average equivalent circle diameter between 0.6 µm to 1.0 µm for the retrieved UHMWPE particles in the synovia fluid obtained from patients who had total knee arthroplasty. Comparing particles from highly crosslinked and conventional UHMWPE, the particle size revealed no significant difference in the studies of Hinarejos et al. [159] and Minoda et al. [158], whereas Iwakiri et al. [160] found particles from highly crosslinked material with half the size of the particles from conventional UHMWPE. Analysis of aspirated synovia fluid was also used by Orita et al. [161] and he found 3.4 years postoperatively an average equivalent circle diameter (standard deviation) of 0.49 (0.10) µm for vitamin E infused highly crosslinked UHMWPE and 1.46 (0.32) µm for conventional UHMWPE (GUR1050, gamma sterilized). Five years postoperatively, Kim et al. [162] found an average equivalent circle diameter (standard deviation) of 0.59 (0.05) µm and 0.52 (0.03) µm in aspirated synovia fluid from patients treated with a ram-extruded and ethylene oxide-sterilized polyethylene articulating against oxidized zirconium and CoCr femoral component, respectively. In this study, patients younger than 60 years at the time of surgery were treated bilaterally with a CoCr femur component on one side and an oxidized zirconium femur component on the opposite side. For the same implant design, Minoda et al. found one year postoperatively an average equivalent circle diameter (standard deviation) of 0.8 (0.3) µm for the oxidized zirconium implant and 0.6 (0.1) µm for the CoCr implant. Bigger particles in this study compared to the clinical findings are presumably related to the application of highly demanding activities, whereas in patient behaviour the relation between level walking and stair climbing is only ten to one [74].

The influence of vitamin E on the biological tolerance of UHMWPE particles was analysed in cell culture tests and animal studies. Bladen et al. [163] found reduced osteolytic mediators expression in lipopolysaccharide stimulated peripheral blood mononuclear cells in the presence of vitamin E, whereas particles from virgin UHMWPE stimulated a higher cytokine expression comparted to particles from UHMWPE with vitamin E. Bone protective markers were increased and bone erosive markers were reduced for UHMWPE particles with vitamin E compared to particles without vitamin E in cell studies with human osteoblasts of Galliera et al. [164,165], concluding that Vitamin E-blended UHMWPE induced an osteoimmunological response, thereby reducing the aseptic loosening of the implants and therefore improving the longevity of total joint replacement. Additionally, for a crosslinked and vitamin E diffused UHMWPE, a bactericidal effect against S. aureus was observed by Chen et al. [166] in cell culture tests with murine RAW 264.7 macrophages and in a murine calvaria animal test. Less osseous resorption and lower inflammatory reaction for highly crosslinked and vitamin E infused UHMWPE particles versus highly crosslinked UHMWPE particles was observed by Huang et al. [167] in a murine calvaria particle-induced animal model. The difference in the particle size for the two materials, even both were in the submicron size range, made it difficult to relate the different biological response to the difference in materials or particle sizes. Less osteolytic potential of particles from highly crosslinked and vitamin E diffused UHMWPE compared to particles from highly crosslinked UHMWPE is reported by Bichara et al. [168] and Chen et al. [166] using the same animal model. Compared to conventional UHMWPE particles no significant difference in the biological response was observed for the highly crosslinked and vitamin E infused UHMWPE particles [167,169]. All these information indicate the reduced osteolytic potential of particles from highly crosslinked UHMWPE infused or blended with vitamin E compared to highly crosslinked UHMWPE particles.

## 5. Conclusions

To predict the clinical wear behaviour of new implant materials, the second and third postoperative decade should be accounted with regards to the possibility of material degradation due to the stress accumulation of high load and high flexion activities. Extended accelerated aging for six weeks and the simulation of highly demanding activities can simulate the long-term retrieval findings for chemical, mechanical and tribological properties on crosslinked UHMWPE gliding surfaces. The same test conditions show minor impact on OI values of Vitamin E blended UHMWPE without further consequences on mechanical and tribological properties. Vitamin E can supress or postpone the chemical and mechanical degradation of UHMWPE.

OI is not a good predictor for the mechanical behaviour in UHMWPE without initial free radicals (GUR1020). In these materials, the free radicals are induced by mechanical loading. Subsequently, the oxidation can propagate and the mechanical properties degrade. Nevertheless, for GUR1020X and GUR1020XE, the OI after 6 weeks artificial aging according to ASTM F2003 [49] reflects retrieval findings. Only specimen group GUR1020X exceeded OI values that indicate an effect on the wear and mechanical properties [86], whereas two weeks artificial aging (GUR1020X2w) seems not enough to generate an OI similar to cross-linked UHMWPE long term retrievals. The undetectable cross-linking after 6 weeks aging for GUR1020 is explained based on the intended material condition and for GUR1020X on the perpetuate radical release and chain scission during the oxidation process. The cross-link density after 6 weeks of artificial aging for GUR1020X2w and GUR1020XE are in the expected range based on retrieval observations and a laboratory study. Comparing GUR1020X2w and GUR1020X, it is obvious that increased aging time leads to reduced mechanical properties for the cross-linked material. Additionally, the mechanical properties of virgin UHMWPE (GUR1020) are affected by the six weeks artificial aging. Nevertheless, the extended artificial aging provoke a heavier effect on virgin UHMWPE than reported for long term retrievals made from comparable materials, whereas the long-term aging simulates the decrease of the mechanical properties of cross-linked UHMWPE. The consequence of these changes of the mechanical properties are observed during the wear test as mechanical damage (cracks and delamination) for the specimen groups GUR1020 and GU1020X and the absence of fatigue failure for specimen groups GUR1020X2w and GUR1020XE. The wear rate for the latter two materials is well below of currently established wear rates and indicates a tribological behaviour with a good clinical perspective. The distribution of the wear and creep for specimen groups GUR1020X2w and 1020XE is consistent within and between the tested specimen groups. The comparison to retrievals has limitations due to variabilities in design, implant positioning and patient variabilities like activity grade or BMI. Nevertheless, the penetration rates of the tests (GUR1020X2w, GUR 1020XE) are below retrieval values. The particles released during the test indicate no increase in biological activity compared to the in vitro studies or clinically results for established materials. Furthermore, the vitamin E content in the particles released from GUR1020XE offers a good perspective regarding the reduction of septic and aseptic loosening.

## 6. Disclosure Policy

Five of the authors (JS, BF, ALPR, CS, TG) are employees of Aesculap AG Tuttlingen a manufacturer of orthopedic implants. One of the authors (GB) got institutional research funding for analysis of kinematic data. One of the authors (PB) got institutional research funding for analysis of the cross-link density.

## Figures and Tables

**Table 1:** Description of the four groups of specimens used in this study. All specimens were artificially aged for two or six weeks according to ASTM F2003 [49]. For each wear test group, 3 tested specimens and one loaded soak control specimen were used according to ISO 14243-1 [66].

Material	Irradiation Dose	Irradiation Type	Aging Time	Specimen Description
GUR1020X	75 kGy	Gamma	2 weeks	GUR1020X2w
GUR1020X	75 kGy	Gamma	6 weeks	GUR1020X
GUR1020	0	---	6 weeks	GUR1020
GUR1020XE	80 kGy	E-beam	6 weeks	GUR1020XE

**Table 2:** *p*-Values (non parametric ANOVA, Kruskal-Wallis, significant for *p* < 0.05) after comparing the OI values between the specimen groups at the same cutting depth. The global *p*-value of the ANOVA was *p* < 0.0001. The *p*-values from the post-hoc test are reported and highlighted with bold numbers if significant.

Cutting Depth [mm]	Material	GUR1020X2w	GUR1020X	GUR1020
0.0	GUR1020X	**0.000000**		
	GUR1020	0.233113	**0.001802**	
	GUR1020XE	**0.002788**	0.175317	0.908496
0.1	GUR1020X	**0.000000**		
	GUR1020	**0.001240**	0.267626	
	GUR1020XE	0.244172	**0.001441**	0.576584
0.2	GUR1020X	**0.000014**		
	GUR1020	**0.029567**	0.334696	
	GUR1020XE	1.000000	**0.000008**	**0.020569**
0.3	GUR1020X	**0.000060**		
	GUR1020	0.073330	0.334696	
	GUR1020XE	1.000000	**0.000002**	**0.007362**
0.4	GUR1020X	**0.000783**		
	GUR1020	1.000000	**0.037405**	
	GUR1020XE	1.000000	**0.000005**	0.175317
0.5	GUR1020X	**0.000002**		
	GUR1020	**0.011665**	0.292953	
	GUR1020XE	1.000000	**0.000055**	0.081654
1.0	GUR1020X	**0.000003**		
	GUR1020	**0.019339**	0.222483	
	GUR1020XE	1.000000	**0.000078**	0.137023

**Table 3:** *p*-Values (ANOVA, significant for *p* < 0.05) after comparing the OI values within the specimen groups at different cutting depth. The global *p*-value of the ANOVA was *p* < 0.0001 for GUR1020X and GUR1020XE. The *p*-values of the post-hoc test are reported and highlighted with bold numbers if significant. For specimen group GUR1020X2w (global *p* = 0.0136) and GUR1020 (global *p* = 0.9452) no significance was detected with the post-hoc test. The arrows indicate that the first line and the second row in the table are cutting depth.

Cutting Depth [mm] ↓ →		0.1	0.2	0.3	0.4	0.5	1.0
GUR1020X	0.0	0.236339	0.999980	0.705326	0.212038	**0.025989**	0.163247
	0.1		0.374179	**0.002347**	**0.000096**	**0.000003**	**0.000058**
	0.2			0.536987	0.120274	**0.011614**	**0.088948**
	0.3				0.984783	0.675876	0.968948
	0.4					0.984510	1.000000
	0.5						0.993461
GUR1020XE	0.0	**0.000000**	**0.000000**	**0.000000**	**0.000000**	**0.000000**	**0.000000**
	0.1		**0.045180**	**0.001683**	**0.000264**	**0.002330**	**0.000087**
	0.2			0.958683	0.774330	0.973910	0.602857
	0.3				0.999201	1.000000	0.990175
	0.4					0.997912	0.999970
	0.5						0.982393

**Table 4:** *p*-Values (ANOVA) after comparing the results of the mechanical properties for the different specimen groups. The global *p*-value of the ANOVA for all mechanical parameters was *p* < 0.0001. The *p*-values from the post-hoc test are reported and highlighted with bold numbers if significant.

Significant for *p* < 0.05		GUR1020X	GUR1020	GUR1020XE
Work to failure [mJ]	GUR1020X2w	**0.000017**	**0.000079**	0.901156
	GUR1020X		0.901065	**0.000079**
	GUR1020			**0.000393**
Peak load [N]	GUR1020X2w	**0.034103**	0.126024	0.999652
	GUR1020X		0.917506	**0.042128**
	GUR1020			0.151105
Ultimate load [N]	GUR1020X2w	**0.000000**	**0.000000**	0.869565
	GUR1020X		0.688361	**0.000000**
	GUR1020			**0.000000**
Displacement at ultimate load [mm]	GUR1020X2w	**0.000014**	**0.000077**	0.961666
	GUR1020X		0.87641	**0.000005**
	GUR1020			**0.000026**

## Data Availability

The raw processed data required to reproduce these findings cannot be shared at this time as the data also forms part of an ongoing study.
